# A prospective, randomized, open-label trial of early versus late povidone-iodine gargling in patients with COVID-19

**DOI:** 10.1038/s41598-022-24683-8

**Published:** 2022-11-28

**Authors:** Akifumi Matsuyama, Hanayuki Okura, Shyoji Hashimoto, Toshio Tanaka

**Affiliations:** 1 Center for reverse Translational Research, Osaka Habikino Medical Center, Osaka Prefectural Hospital Organization, 3-7-1 Habikino, Habikino, Osaka 583-8588 Japan; 2 Center for Clinical Research, Osaka Habikino Medical Center, Osaka Prefectural Hospital Organization, Habikino, Japan; 3 Osaka Habikino Medical Center, Osaka Prefectural Hospital Organization, Habikino, Osaka Japan; 4 Present Address: Japan Mutual Aid Association of Public School Teachers, Kinki Chuo Hospital, Osaka, Japan

**Keywords:** Microbiology, Health care, Medical research

## Abstract

Povidone-iodine (PVP–I) is a broad-spectrum antiseptic reagent that has been used for over 50 years. The purpose of this study is to look into the effect of gargling with PVP–I gargling on virus clearance and saliva infectivity in COVID-19. A prospective, randomized, open-label trial of intervention with PVP–I was conducted at three quarantine facilities in Osaka, Japan, enrolling adolescents and adults with asymptomatic-to-mild COVID-19. Patients were randomly allocated to the early and late intervention group at a 1:1 ratio. The early group gargled with PVP–I from days 2 to day 6; the late group gargled with water first, then with PVP–I from day 5 after sampling till day 6. The primary and secondary endpoints were viral clearance for SARS-CoV-2 using RT-qPCR at days 5 and 6, respectively, and the investigational endpoint was saliva infectivity clearance on day5. We enrolled 430 participants, with 215 assigned to each group, and 139 in the early group and 140 in the late had a positive saliva RT-qPCR test on day 2. On day 5, the early group had a significantly higher RT-qPCR negative rate than that of the late group (p = 0.015), and the early had a significantly higher clearance rate of infectivity (p = 0.025). During the PVP–I intervention, one participant reported oropharyngeal discomfort. Gargling with PVP–I may hasten virus clearance and reduce viral transmission via salivary droplets and aerosols in patients with asymptomatic-to-mild COVID-19. (Clinical trial registration numbers: jRCT1051200078 and dateof registration: 24/11/2020).

## Introduction

The salivary SARS-CoV-2 viral load is thought to play a significant role in the accelerated transmission of coronavirus disease 2019 (COVID-19)^1^. A reduction in salivary viral load is thought to suppress transmission^2^, and some studies have suggested that mouthwash and/or gargling with povidone-iodine (PVP–I) may have an antiseptic effect for SARS-CoV-2, reducing its viral load^3^. However, it is unknown whether gargling with PVP–I would eliminate salivary viral load and infectivity in COVID-19 patients in vivo.

PVP–I is a polyvinylpyrrolidone (PVP) and iodine complex that has an antiseptic effect by releasing iodine. Its mechanism of action involves the use of iodine to oxidize microbial components. PVP–I has previously been shown to have an antiseptic effect on SARS-CoV and MERS in vitro^4,5^, and it was also effective against SARS-CoV-2^6^. A single in vitro gargle with PVP–I reduced the salivary viral load in two of four patients with a high salivary viral load^7^. Although recent studies have confirmed the short-term effects of reducing salivary viral load in a small number of patients with COVID-19 ^8^, there have been no prospective randomized intervention studies involving PVP–I gargling.

The purpose of this study was to see if gargling with PVP–I will reduce viral load and SARS-CoV-2 infectivity in patients with COVID-19.

## Materials and methods

### Study design

This was a prospective, randomized, and open-label trial that looked at the efficacy and safety of gargling with PVP–I in asymptomatic-to-mild COVID-19 patients (aged ≥ 16 years) quarantined in three different locations. The protocol was approved by the Medical Center Clinical Research Review Board (CRB5200005), which has been establishe in Osaka General Medical Center, Osaka Prefectural Hospital Organization, Osaka, Japan. The first registration date is 24/11/2020 with the Japan Registry of Clinical Trials (number: jRCT1051200078). The study has been performed in accordance with the relevant guidelines and regulations and all study participants provided written informed consent.

### Patients and randomization

The study has performed from 24/11/2020 to 22/4/2022. From 30/11/2020, to 17/3/2021, participants were recruited from three quarantine facilities in Osaka, Japan. On 22/3/2021, the follow-up was completed. The following inclusion criteria were used to select subjects: (1) age ≥ 16 years on the date of consent acquisition and with parental consent for those aged 16–19 years; (2) either sex; (3) quarantined at an accommodation facility but not inpatient status; (4) meet all of the following requirements: asymptomatic-to-mild COVID-19 patients with at least one (RT-PCR) test positive for SARS-CoV-2 (either pharyngeal swab, nasopharyngeal swab, or saliva test), capable of mouthwash and gargling, and willing to be quarantined for ≥ 6 days; and (5) voluntarily provided written consent to participate in the trial. The following were exclusion criteria: (1)being on receiving thyroid hormone, (2) having an iodine allergy, (3) being pregnant, (4) lactating female, and (5) already mouth washing or gargling with PVP–I prior enrollment.

Patients were randomly assigned at a 1:1 ratio with Viedoc4 EDC ststem to either the early interventional group (started mouthwash and gargling with PVP–I on day 2 after saliva sampling at the time of waking up) or the late interventional group (started mouthwash and gargling with PVP–I on day 5 after sampling of saliva at the time of waking up); accommodation facility, age, and gender were used as allocation adjusting factors. Because no placebos were available, the study was conducted in an open-label.

### Intervention procedure

Table 1 depicts the saliva sampling and the intervention schedule. Mouth washing once and gargling twice with 20 mL of a 15-fold diluted solution containing PVP–I Gargle Solution 7% (Meiji Seika Pharma Co., Ltd., Tokyo, Japan) or with the same volume of water constitute the intervention. From day 2 to day 6, the early interventional group was instructed to perform the PVP-1 intervention four times per day (before saliva sampling at the time of waking up, before lunch, before supper, and before sleeping). In the late interventional group, the intervention was performed with water from days 2 to 4, and then with PVP–I from days 5 following saliva sampling till day 6.

**Table 1:** The research schedule.

		Pre	Day 1 before enrollment	Day 1 after enrollment	Day 2	Day 3	Day 4	Day 5	Day 6	At the time of early termination	At the time of research discontinuation
Obtaining informed consent		●									
Enrollment			●								
Baseline			●								
Saliva sample	Before mouthwash and gargling				●	●	●	●	●		
Observation	AM				●	●	●	●	●	●	●
	PM			●	●	●	●	●			
Early interventional group											
Mouthwash and gargling with PVP-I	Upon waking up and before sampling of saliva				●	●	●	●	●		
	Before lunch				●	●	●	●			
	Before supper				●	●	●	●			
	Before sleeping				●	●	●	●			
Late interventional group											
Mouthwash and gargling with water	Upon waking up and before sampling of saliva				●	●	●				
	Before lunch				●	●	●				
	Before supper				●	●	●				
	Before sleeping				●	●	●				
Mouthwash and gargling with PVP-I	Upon waking up and before sampling of saliva							●	●		
	Before lunch							●			
	Before supper							●			
	Before sleeping							●			

●: Required.

Body temperature, presence or absence of physical condition changes, cough, suffocation, runny nose, stuffy nose, sore throat, nausea/vomiting, red-eye, headache, dullness, joint muscle pain, diarrhea, light-headedness/consciousness, miscellence, deemed necessary.

### Measurement

The KANEKA Direct RT-qPCR Kit “SARS-CoV-2” was used for RT-qPCR testing at a centralized study laboratory, as directed by the manufacturer (Kaneka Corporation, Tokyo, Japan). A LightCycler 480 II was used to monitor amplified products (Roche Diagnostics, Basel, Switzerland). The SARS-CoV-2 virus was set to Ct = 40, as recommended by the manufacturer.

The saliva was tested for viral infectivity in a biosafety level 3 laboratory at the Osaka Habikino Medical Center. Vero E6 TMPRSS2 cells (JCRB1819) were obtained from JCRB bank at the National Institutes of Biomedical Innovation, Health, and Nutrition, Osaka, Japan. The cells were cultured according to the institution’s instruction and seeded into 96 well plates with 100 µL of medium and cultured for a day. Then, 10 µL of each salivary sample was added. The cells were monitored for cytopathic effect (CPE) after a 3-day incubation period, and SARS-CoV-2 infectivity was confirmed when CPE was detected in inoculated wells.

### Observation schedule

The study’s observation schedule is shown in Table 1. Salivary samples were collected daily from days 2 to 6 upon waking up in the morning, before mouthwash and gargling; these were centrifuged to remove debris before being analyzed using RT-qPCR and CPE. During the study, the participants’ body temperature and physical conditions (presence or absence of changes, cough, suffocation, nasal congestion, stuffy nose, sore throat, nausea/vomiting, red-eye, headache, dullness, joint muscle pain, diarrhea, and light-headedness/loss of consciousness) were recorded twice a day.

The primary efficacy endpoint of this study was the clearance rate of SARS-CoV-2 viral load determined by RT-qPCR of saliva samples on day 5. On day 5, the clearance rate in each group was calculated by dividing the number of RT-qPCR-negative participants on day 5 by the number of RT-qPCR-positive participants on day 2. The 95% confidence interval (CI) was also computed. The chi-square test was used to compare the clearance rates of the early groups. The viral clearance rate of saliva samples at day 6, was calculated and analyzed using the same methods as for the primary endpoint. Safety was assessed based on adverse events that occurred during the study period by aggregating the number of cases and the number of cases by events in each group. The rate of SARS-CoV-2 viral infectivity to Vero E6 TMPRSS2 cells in saliva samples at day 5 was the investigational endpoint of efficacy. On day 5, the rate in each group was calculated by dividing the number of infectivity-positive participants on day 5 by the number of RT-qPCR-positive participants on day 2. The 95% CI was computed. The chi-square test was used to compare rates of the early and late groups.

In this study, datasets for intention-to-treat (ITT) and safety analysis (SAF) were determined and analyzed. In the efficacy evaluation, all subjects who used the PVP–I was included in the ITT, whereas all subjects who used the PVP–I were analyzed in the safety evaluation. The name of the adverse event was changed to MedDRA/J ver. 24.0 and used for analysis.

### Statistical analysis

The sample size was calculated based on the assumption that viral clearance of saliva could be observed by day 5 in 60% of participants in the early interventional group with PVP–I and 40% in the late interventional group. With a power of ≥ 90% and a two-sided significance level of 0.05, this study required 203 participants in each group. To account for a 10% dropout rate, each group required 220 subjects. SAS software, version 9.4 was used for statistical analyses (SAS Institute, Cary, NC, USA).

### Ethical approval

The study was centrally approved by the certified Medical Center Clinical Research Review Board (CRB5200005) which has been establishe in Osaka General Medical Center, Osaka Prefectural Hospital Organization, Osaka, Japan and was registered on 24/11/2020 with the Japan Registry of Clinical Trials (number jRCT1051200078).

## Results

### Patients

**Figure 1:**
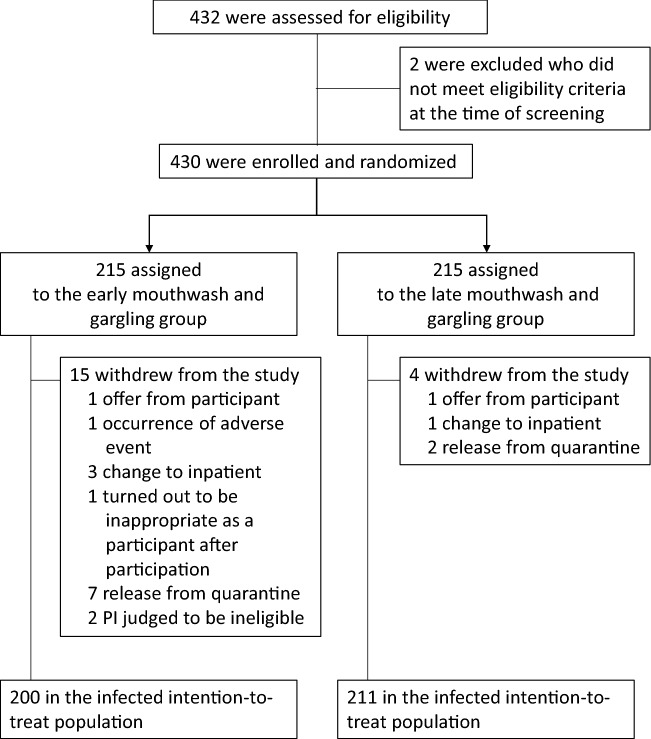
Patient enrollment and intervention assignment.

The first case was filed on 30/11/2020, and the last case was completed on 22/3/2021. The interventional groups were assigned to 432 subjects who gave consent(215 subjects each). Figure 1 depicts participants’ information, 430 were randomized and equally assigned to the interventional groups (215 subjects each). Each intervention group had 215 patients efficacy analysis target population (ITT). The SAF population comprised 215 patients in the early mouthwash and gargling group and 213 in the late group (two participants discontinued before PVP–I intervention). After randomization 15 patients in the early group and four in the late group withdrew from the study. As a result, 411 patients who were part of the ITT population had data related to the study. There were seven patients in the early and two in the late who were untraceable after the quarantine was lifted. In all groups, the implementation rate of mouthwash and gargling was 95% or higher.

Table 2 depicts the patient background in the efficacy analysis population. The early intervention group had a mean age ± standard deviation was 45.2 ± 15.6 years and the late intervention group had a mean age standard deviation of 45.1 ± 15.2 years. In both groups, the proportion of people aged ≥ 50 years or older was the highest (43.4% and 43.7% in the early and intervention groups, respectively). In terms of gender, the early group comprised 56.7% males and 43.3% females. Complications occurred in 23.3% of the subjects in the early group and 25.1%of subjects in the late group. The proportion of subjects with fever during participation was 27.0% in the early group and 33.0% in the late group. The proportion of subjects who had fever during the study was 27.0% in the early group and 33.0% in the late group.

**Table 2:** Participants with COVID-19's baseline demographics and clinical characteristics.

	Early interventional group	Late interventional group	
Demographic and clinical characteristics	n = 215	n = 215	p-value*
**Age (in years)**			
Average (standard deviation)	45.2 (15.6)	45.1 (15.2)	t: 0.960
Median (minimum–maximum)	47.0 (20–79)	47.0 (17–79)	
**Age demographics**			
16–19 years (%)	0 (0.0)	1 (0.5)	x: 0.879
20–29 years (%)	53 (24.7)	50 (23.3)	
30–39 years (%)	21 (9.8)	23 (10.7)	
40–49 years (%)	48 (22.3)	47 (21.9)	
50 years and over (%)	93 (43.3)	94 (43.7)	
**Sex**			
Male (%)	122 (56.7)	121 (56.3)	F: 1.000
Female (%)	93 (43.3)	94 (43.7)	
**Coexisting disease**			
None (%)	165 (76.7)	161 (74.9)	F: 0.736
Yes (%)	50 (23.3)	54 (25.1)	
Hypothyroidism (%)	0 (0.0)	0 (0.0)	
Other (%)	50 (23.3)	54 (25.1)	
**Drug allergy**			
None (%)	210 (97.7)	211 (98.1)	F: 1.000
Yes (%)	5 (2.3)	4 (1.9)	
Povidone iodine (%)	0 (0.0)	0 (0.0)	
Others (%)	5 (2.3)	4 (1.9)	
**Other allergies**			
None (%)	195 (90.7)	201 (93.5)	F: 0.372
Yes (%)	20 (9.3)	14 (6.5)	
Hay fever (%)	13 (6.0)	11 (5.1)	
Allergic rhinitis (%)	4 (1.9)	2 (0.9)	
Food allergies (%)	0 (0.0)	0 (0.0)	
Others (%)	3 (1.4)	2 (0.9)	
**Subjective fever**			
None (%)	157 (73.0)	144 (67.0)	F: 0.207
Yes (%)	58 (27.0)	71 (33.0)	
**Days from fever to randomization**			
Number of examples	58	71	
Average value (standard deviation)	− 2.5 (1.4)	− 2.5 (1.8)	t: 0.980
Median (minimum–maximum)	− 3.0 (− 8–0)	− 2.0 (− 13–0)	
**Other symptoms**			
None (%)	115 (53.5)	100 (46.5)	F: 0.177
Yes (%)	100 (46.5)	115 (53.5)	
**Days from the onset of other symptoms to randomization**			
Number of examples	98	113	
Average value (standard deviation)	− 2.5 (1.6)	− 2.1 (1.4)	t: 0.072
Median (minimum–maximum)	− 2.0 (− 8–0)	− 2.0 (− 9–0)	
**Mouthwash or gargling with PVP-I until the quarantine**			
None (%)	215 (100.0)	215 (100.0)	F: –
Yes (%)	0 (0.0)	0 (0.0)	
**Accommodation for quarantine**			
Hotel S	91 (42.3)	90 (41.9)	x: 0.995
Hotel A	30 (14.0)	30 (14.0)	
Hotel W	94 (43.7)	95 (44.2)	

*t: Student’s t-test, F: Fisher’s exact test, x: chi-square test.

### Efficacy

The clearance rate of SARS-CoV-2 in saliva on day 5 was 34.5% (95% CI: 26.68–43.06%) in the early mouthwash and gargling group and 21.4% (95% CI: 14.95–29.16%) in the late group for the primary endpoint. The former gargled with PVP–I for 3 days, while the latter gargled with water (but not PVP–I). The viral clearance rate of SARS-CoV-2 was significantly higher for mouthwash and gargling with PVP–I for 3 days than for mouthwash and gargling with water (p = 0.015) (risk ratio: 1.612, 95% CI: 1.090–2.383) (Table 3). RT-PCR data of each participant were shown in Supplementary Table 1. Moreover, the viral clearance rate on days 5 and 6 was significantly higher in the early group than in the late group among those aged 40–49 years (p = 0.009 and p = 0.009, respectively) in the subgroup analysis by gender and age. CPE data of each participant were shown in Supplementary Table 2.

**Table 3:** Changes in the clearance rate of SARS-CoV-2 in saliva.

Evaluation date	Interventional group	ITT	Number of evaluated subjects^a^	Positive	Negative	95% confidence interval	p-value
			n (%)	n (%)	n (%)		
Day 2	Early group	198	–	139 (70.2)	59 (29.8)	23.52–36.69	0.485
	Late group	209	–	140 (67.0)	69 (33.0)	26.68–39.84	
Day 3	Early group	–	139 (70.2)	108 (77.7)	31 (22.3)	15.68–30.14	0.041
	Late group	–	139 (66.5)	121 (87.1)	18 (12.9)	7.86–19.69	
Day 4	Early group	–	139 (70.2)	110 (79.1)	29 (20.9)	14.44–28.57	0.687
	Late group	–	140 (67.0)	108 (77.1)	32 (22.9)	16.19–30.71	
Day 5	Early group	–	139 (70.2)	91 (65.5)	48 (34.5)	26.68–43.06	0.015
	Late group	–	140 (67.0)	110 (78.6)	30 (21.4)	14.95–29.16	
Day 6	Early group	–	138 (69.7)	81 (58.7)	57 (41.3)	33.00–49.99	0.441
	Late group	–	136 (65.1)	86 (63.2)	50 (36.8)	28.67–45.45	

^a^Subjects who were positive for COVID-19 on day 2.

Regarding the secondary endpoint, the SARS-CoV-2-clearance rate in saliva on day 6 was 41.3% (95% CI: 33.00–49.99%) in the early mouthwash and gargling group and 36.8% (95% CI: 28.67–45.45%) in the late group. The former gargled with PVP–I for 4 days, while the latter gargled with water for 3 days and then with PVP–I for 1 day. There were no significant differences between the two groups (Table 3). During the study period, there was no difference between the two groups in COVID-19-related subjective and objective symptoms.

The infectivity ratio of saliva on day 5 compared to SARS-CoV-2 PCR positive participants (139 in the early group and 140 in the late group, respectively) was 2.9% in the early gargling group and 9.3% in the late group (p = 0.025) (Table 4).

**Table 4:** SARS-CoV-2 viral infectivity rate on day 5 compared to RT-qPCR positives on day 2.

Group	ITT	Virus detected in (n, day 2) by RT-qPCR	Number of subjects^a^	CPE-Positive	CPE-Negative	95% confidence interval	p-value
			(day 5)	n (%)	n (%)		
Early interventional group	198	139	139	4 (2.9)	135 (97.1)	92.80–99.21	0.025
Late interventional group	209	140	140	13 (9.3)	127 (90.7)	84.64–94.96	

^a^Subjects who tested positive for COVID-19 on day 2 and were retested on day 5 were included in the study.

### Safety

The incidence of adverse events in the enrolled population was investigated by severity and casualty. During the study period, only one adverse event (oropharyngeal discomfort) was observed in the early interventional group (Table 5). Although a causal relationship between the oropharyngeal discomfort and the PVP–I intervention was identified in the case, and the patient was removed from the study, the severity was grade 1 and resolved 2 days after onset. In this study, no deaths or serious adverse events were observed.

**Table 5:** The occurrence of adverse events (SAF).

	Early interventional group		Late interventional group		Total	
	n = 214		n = 214		n = 428	
Item	Number	Number of subjects (%)	Number	Number of subjects (%)	Number	Number of subjects (%)
All adverse events	1	1 (0.5)	0	0 (0.0)	1	1 (0.2)
**Grade**						
1	1	1 (0.5)	0	0 (0.0)	1	1 (0.2)
2	0	0 (0.0)	0	0 (0.0)	0	0 (0.0)
3 or more	0	0 (0.0)	0	0 (0.0)	0	0 (0.0)
**Causal relationship**						
Not relevant	0	0 (0.0)	0	0 (0.0)	0	0 (0.0)
Related	1	1 (0.5)	0	0 (0.0)	1	1 (0.2)
Serious adverse events	0	0 (0.0)	0	0 (0.0)	0	0 (0.0)

## Discussion

This randomized clinical trial of asymptomatic-to-mild COVID-19 patients found a significant difference in the viral load and infectivity at day 5 between those who gargled with PVP–I for 3 days those who gargled with water. From days 3 to 6, the early interventional group has a higher viral clearance than the late group. Moreover, after 1 day with the PVP–I intervention, the viral clearance rate in the late group was approaching that of the early interventional group on day 6. The only adverse event observed during the study period was oropharyngeal discomfort, and the safety of PVP–I intervention in patients with asymptomatic or mild COVID-19 was confirmed.

PVP–I is a broad-spectrum antiseptic reagent that has been used for over 50 years and is effective in vitro against SARS-CoV and MERS^9^. Because SARS-CoV-2 is a coronavirus, the effect of PVP–I on the SARS-CoV-2 in vitro has been studied^10^, and PVP–I mouthwash were assumed to reduce the burden of SARS-CoV-2 virus in saliva^11^. Because saliva-derived droplets and aerosols have been linked to SARS-CoV-2 transmission^1^, it has been investigated whether salivary viral load could be reduced by PVP–I intervention. Small studies in vivo have shown that the intervention with PVP–I resulted in a significant reduction in viral load in half of their participants (n = 4), which at least 3 h^7^. In contrast, there is a controversial report in which the participants’ salivary Ct values showed no significant differences at 5 min, 3 h, and 6 h timepoints^8^. However, previous studies have studies only looked at a single mouthwash with PVP–I, so, we wondered if antiseptic effects could be obtained through continuous PVP–I intervention. SARS-CoV-2 infection caused the rapid onset of upper respiratory tract infection with high peak viral loads, as reported by SARS-CoV-2, and the virus could be detected in throat swabs earlier than in the nose after inoculation^12^. The viral clearance effect of gargling with PVP–I on salivary viral load and of SARS-CoV-2 infectivity was demonstrated in this study. Hence, the antiseptic effect of PVP–I may reduce viral load in saliva and viral transmission in the oral cavity of COVID-19.

Patients have been reported to suffer from adverse events due to allergic reactions to iodine in rare but significant cases; PVP–I is also contraindicated in patients with thyroid diseases^13^. We excluded subjects who were (1) taking a thyroid hormone, (2) allergic to iodine, (3) pregnant, (4) lactating females, and (5) using mouthwash or gargling with PVP–I before participation. During the PVP–I intervention in our study, only one participant (0.5% of the early group) in the safety population reported throat discomfort as an adverse event. Although none of the 6692 patients who came to the hospital for office ENT consultations reported any serious side effects or allergic reactions to 0.5% PVP–I nasal drops and gargles^14^, it is important to consider the potential adverse events and contraindications when using PVP–I in clinical practice.

During the COVID-19 pandemic prophylactic use of PVP–I was proposed and recommended. There are two concerns: the first is who might be able to avoid infection through intervention, and the second is who should be treated. The American Dental Association (ADA) has issued the ADA Interim Guidance for Minimizing Risk of COVID-19 Transmission, which recommends PVP–I as a preprocedural mouthwash^15^. The Australian Dental Association has also issued the ADA COVID-19 Risk Management Guidance, which states that all patients should perform a preprocedural mouthwash with PVP–I for 20 s before treatment^16^. According to the above guidelines, healthcare providers should benefit from PVP–I intervention. The report that all healthcare providers who collectively consulted 6692 ENT patients who escaped from COVID-19 infection could reinforce these recommendations^13^. Some ongoing studies are looking into the possibility of healthcare providers being free from COVID-19 by using PVP–I prophylaxis^17,18^. An open-label parallel randomized controlled trial in Singapore recently found significant absolute risk reductions in SARS-CoV-2 infection after PVP–I throat spray in healthy young people and migrant workers ^19^. These findings suggest that PVP–I intervention may be a prophylactic effect in a healthy population at high risk for COVID-19. Conversely, reducing transmissions from COVID-19 patients is important because only 2% of SARS-CoV-2-positive individuals have been reported to carry 90% of the virus circulating in communities^20^. The efficacy of PVP–I intervention on the infected patient was demonstrated in our study. More findings should consider targeting either diseases spreaders or healthy populations to control disease transmission and overcome the pandemic.

Several limitations are noteworthy in our study. First, the study was conducted in three quarantine facilities, all of which were located on the same site. The second limitation is that the open-label study design may have resulted in biased management and assessment, in which, there is a possibility that the participants garging with water but PVP-I increased the frequency of gargling by setting it as an open label study. Third, only included only asymptomatic or mildly ill patients; our findings cannot be generalized to patients with moderate-to-severe COVID-19. The fact that only asymptomatic-to-mildly ill patients were recruited in an underestimation or overestimation of the potential clinical benefits of PVP–I in individuals. Finally, the primary and secondary endpoints were only assessed using RT-qPCR, and it is unknown whether mouthwash or PVP–I gargle had any effect on SARS-CoV-2-producible cells (Supplementary Tables 1 and 2).

Mouthwash and gargling with PVP–I for 3 days improved viral clearance in patients with asymptomatic or mild COVID-19 in this randomized trial. Furthermore, After 3 days of mouthwash and gargling with water, mouthwash and gargling with PVP–I in 1 day could catch up in terms of viral clearance rate to those who underwent 4 days of gargling with PVP–I. This demonstrates that the late intervention with PVP–I is not inferior to early intervention, indicating that it is recommended to gargle with PVP-I even in the late phase of the disease. Our findings suggest that PVP–I reduce viral load in the saliva of the asymptomatic-to-mild COVID-19 patients. COVID-19 transmission could be reduced if PVP–I intervention could convert super-spreaders into moderate, mild, or non-spreader patients. Further research should be conducted to determine whether the effect translates into COVID-19 prevention in the general population and the treatment of individuals with the disease in the COVID-19 era.

## Supplementary Information

Supplementary Table 1.

Supplementary Table 2.

## Data Availability

The datasets used and/or analysed during the current study available from the corresponding author on reasonable request.
